# Clinical and radiographical evaluation of the healing of large periapical lesions using triple antibiotic paste, photo activated disinfection and calcium hydroxide when used as root canal disinfectant

**DOI:** 10.4317/jced.51324

**Published:** 2014-07-01

**Authors:** Dexton A. Johns, Jolly M. Varughese, Kunjamma Thomas, Aby Abraham, Elizabeth P. James, Ramesh K. Maroli

**Affiliations:** 1 MDS, Assistant Professor Department of Endodontics. KMCT dental college, calicut, kerala, India; 2 MDS, Principal, Department of Endodontics. Govt dental college, trivandrum kerala, India; 3 MDS, Principal,Department of Endodontics. KMCT dental college, Calicut, kerala, India; 4 MDS, Department of orthodontics. Govt dental college, kottayam, kerala, India; 5 MDS, Assistant Professor Department of Endodontics. Govt dental college, calicut, kerala, India; 6 MDS, Professor ,Department of Endodontics. Govt dental college ,calicut, kerala, India

## Abstract

Objectives: To evaluate clinically and radio graphically, the healing following nonsurgical treatment of periapical lesions when Photo Activated Disinfection(PAD), triple antibiotic paste and calcium hydroxide was used as root canal disinfectant. Material and Methods: Sixty patients (20 for PAD, 20 for triple antibiotic paste, 20 for calcium hydroxide) with periapical lesions in the maxillary and mandibular anterior region were selected from the outpatient section of the Department of Conservative Dentistry & Endodontics, Govt. Dental College, Kozhikode to participate in this study. The patients were selected with a preoperative score of 4 or 5. There were no significant differences for the PAI Scores between the three groups at the start of the experiment .Intracanal disinfection was done in the three groups followed by obturation. The patients recalled at 3,6,12,18 months interval. Results: At 18 months follow up 15 % of cases failed in calcium hydroxide group,5% in triple antibiotic paste and no failure cases were seen in PAD group. Success criteria were divided into strict and loose, while the former had statistically significant p value the latter did not. Kruskal-Wallis Test showed an increased mean value for PDT and a significant change in p value. Bonferroni post hoc test was done to compare if there is any significant change between groups. Only significant change was found between calcium hydroxide and photoactivated disinfection . Conclusion: PAD was more effective intracanal disinfectant at 6,12 and 18 months.

** Key words:**Calcium, hydroxide, photo activated disinfection, triple antibiotic paste, root canal disinfection

## Introduction

Elimination of microorganisms from the root canal system is one of the objectives of the root canal treatment and has a substantial effect on the treatment outcome. Accepted treatment procedures to eliminate the infection include a combination of chemo-mechanical debridement, application of an inter appointment dressing containing an antimicrobial agent and finally sealing of the root canal. Unfortunately, micro organisms may remain after conventional canal preparation, either within the dentinal tubules or bound within the apical dentin plug. Therefore for complete eradication of infection, the pulpal remnants as well as smear layer should be removed from the root canals.

Some investigators recommend the use of calcium hydroxide as an intracanal dressing in a multiple-visit approach. The environment within the system, however, is such that delivering the medicament and maintaining a high pH homogeneously is a challenge. For these and other reasons, certain microbial species in a limited group of cases do survive and can be responsible for persistent infections (1,2). Thus, the search for a better alternative has led to the discoveries of newer antimicrobial agents.

The Cariology Research Unit of the Niigata University has developed the concept of ‘Lesion sterilization and tissue repair LSTR’ therapy (3,4) that employs the use of a combination of antibacterial drugs for disinfection of oral infectious lesions, including dentinal, pulpal, and periradicular lesions. Repair of damaged tissues can be expected if lesions are disinfected (5). Metronidazole was the first choice because it has a wide bactericidal spectrum against anaerobes (6), which were common in oral sites. However, some bacteria in lesions were resistant to metronidazole and, thus, two other antibacterial drugs, e.g., ciprofloxacin and minocycline, should be mixed with metronidazole (7) in an effort to eliminate all the bacteria. Finally, extensive in vitro and in situ studies have been conducted showing the mixed drugs to be effective against oral bacteria (5,8,9). The disadvantage of this mixture is the discoloration caused by minocycline present in it (10).

In recent years novel antimicrobial approaches to disinfect root canals have been proposed that include Photo Activated Disinfection [PAD]. PAD uses a combination of photosensitising dye, such as Tolonium chloride solution [TC] [synonym Toluidine Blue O], and light of a specific wavelength. This combination using light at 633G2 nm has been shown to kill high numbers of bacteria in planktonic suspension, probably by disruption of the bacterial membrane by short range free radicals or reactive oxygen species. TC is unchanged by the process, which ceases when irradiation stops (11).

The present study was designed to evaluate clinically and radiographically the healing of periapical lesions using triple antibiotic paste, PAD and calcium hydroxide when used as root canal disinfectant.

## Material and Methods

The study is conducted as a prospective clinical trial to compare and evaluate clinical and radiographic healing following nonsurgical treatment of periapical lesions when PDT, triple antibiotic paste and calcium hydroxide were used as root canal disinfectant.Sixty patients [20 for PDT, 20 for triple antibiotic paste, 20 for calcium hydroxide] with periapical lesions in the maxillary anterior region were selected from the outpatient section of the Department of Conservative Dentistry & Endodontics, Govt. Dental College, Kozhikode to participate in this study. The study started in the year 2010 after the approval of the ethical committee of Govt. Medical College, Calicut. Patients were randomly allocated into 3 groups by a random allocation software. One blinded nurse enrolled all participants and assigned them to intervention. The data was statistically analyzed by a statistician who was unaware of the allocated group.

## Inclusion criteria

Patients between 15-30 years of age who had periapical lesions in the maxillary anterior region.

- Exclusion criteria

• All patients who respond positive to allergic patch test [triple antibiotic paste] and drugs.

• Teeth with previous endodontic therapy performed.

• Patients with a history of any systemic diseases.

• Pregnant and lactating women.

• Tooth associated with vertical root fracture and coronal perforation.

• Tooth affected with calcific degeneration.

• Presence of external or internal root resorption.

• Blunderbuss apex.

- Clinical parameters

Clinical details about the treated tooth included: [i] tenderness to pressure and percussion of the tooth, [ii] tenderness to palpation of adjacent soft tissues, [iii] presence of an associated sinus tract or swelling in the adjacent soft tissues, [iv] periodontal probing profile around the tooth, and [v] the type and presence of an adequate coronal restoration and ‘seal’.

- Radiographic parameters

• Radiographs 

Periapical radiographs were taken by a radiologist using the long-cone paralleling technique [Asahi x-ray unit GX-60N; Asahi Roentgen International, Kyoto, Japan]. Kodak DF-57 films [Eastman Kodak, Rochester,NY], which were automatically developed and fixed [Level 360; Flat Co, Kobe, Japan], were used in this study.

• Radiographic examination 

**Figure 1:**
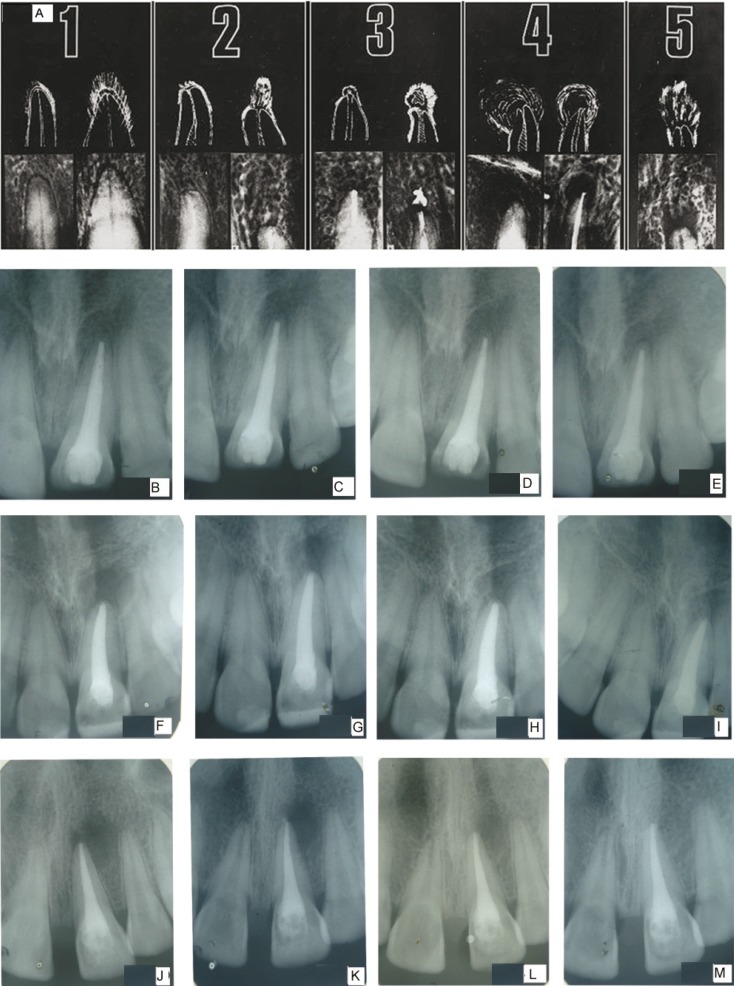
A. Periapical index assessment; b. Calcium hydroxide obturation; c. Calcium hydroxide 6 months; d. Calcium hydroxide 12 months; e. Calcium hydroxide 18 months; f.Triple antibiotic paste obturation; g. Triple antibiotic paste 6 months; h. Triple antibiotic paste 12 months; i. Triple antibiotic paste 18 months; j. Photoactivated disinfection obturation; k. Photoactivated disinfection 6 months; l. Photoactivated disinfection 12 months; m. Photoactivated disinfection 18 months.

Viewing conditions were standardized using a slide viewer [Flash-pak projector; Slidex Co, Tokyo, Japan] with magnification [6.6X]. The periapical status was assessed by using the periapical index [PAI]. Each tooth was assigned to 1 of the PAI scores by using visual references (12) for the 5 categories within the scale (Fig. 1,  Table 1). After scoring the teeth, the results were compared to a gold standard atlas, and Cohen kappa value was [0.81].

**Table 1:**
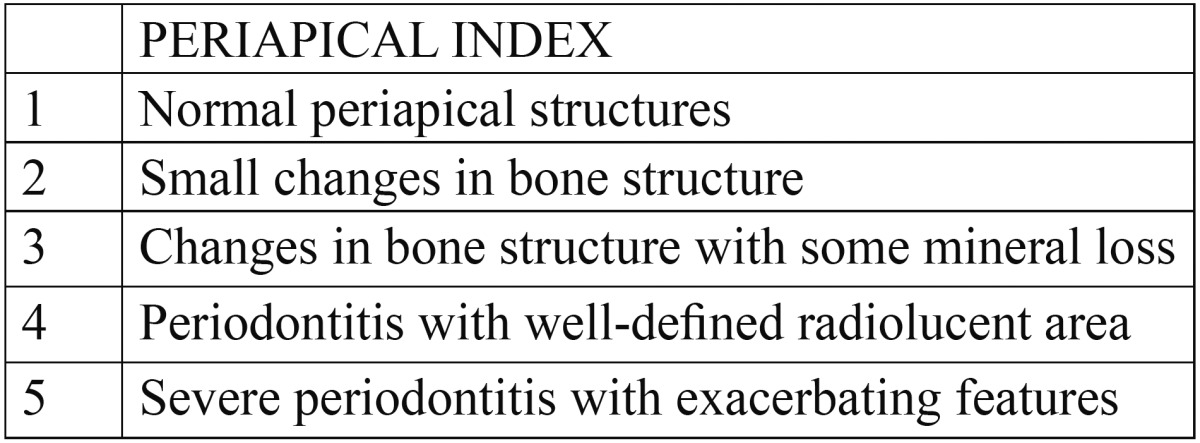
Score Criteria.

According to Landis and Koch [1977] (13), Kappa scores greater than 0.8 indicate “good agreement”.

- Periapical Index

• Favorable 

healed: 3, 4, 5 at IPO [initial pre operative] --> 1-2 at Follow up or 1-2 at IPO -->1-2 at Follow up.

healing: 3, 4, 5 at IPO improves but isn’t --> 1-2 at Follow up.

• Unfavorable not healed/healing

5-3 at IPO stays --> 5-3 at Follow up.

or 1-2 at IPO --> 3, 4, 5 at Follow up.

- Determination of outcome

Treatment success was assessed using two outcome measures. For this part of the study, successful treatment based on strict criteria was defined as absence of pain, clinical evidence of inflammation or swelling and conventional radiographic measures of complete healing/presence of a normal periodontal ligament space.

Successful treatment based on loose criteria was defined as absence of pain, clinical evidence of inflammation or swelling and conventional radiographic measures of complete healing/presence of a normal periodontal ligament space or incomplete healing [if there was reduction in size of the lesion without return to normal periodontal ligament space width].

If a tooth had been extracted because of endodontic problems [persistent pain, swelling, sinus or periapical radiolucent lesion], the treatment was considered failed.

- General Steps

All the cases were treated by one operator using a standardized technique .The access to the pulp chamber was gained and rubber dam was placed. The working length was using electronic apex locator Root ZX [J.Morita MFG. Corporation, Kyoto, Japan] and by files in radiograph. In most cases drainage was performed daily on two to four subsequent appointments until discharge through the canal ceased. The access cavities were sealed with zinc oxide eugenol cement [Dental Products of India,Mumbai,India] after drainage.Intracanal medication and photodynamic therapy was not applied to the canal until active drainage ceased. The root canals were finally instrumented by a conventional step-back technique using K-type files, and copious irrigation with 1% sodium hypochlorite [Nova Dental Products Pvt. Ltd, Mumbai, India], 17%EDTA [B.N. Laboratories,Mangalore,India] and 0.2% chlorhexidine [Vishal Dentocare Pvt.Ltd.,Ahmedabad,India] under rubber dam isolation.

• Group 1

Calcium hydroxide paste [Multical, pulpdent, USA] was used

• Group 2 

Tri antibiotic paste [adapted from Hoshino et al.] containing Ciprofloxacin [Cifran500mg, Ranbaxy Laboratories Ltd., India], Metronidazole [metrogyl 400mg,J.B.Chemicals and Pharmaceuticals Ltd .,India] and Minocycline [Minoz100mg,Ranbaxy Laboratories Ltd.,India] mixed with Macrogol Ointment and Propylene Glycol was used.

• Group 3 

Tolonium chloride 0.01% w/v in aqueous solution was the photosensitizer and 300μm diameter fiber coupled diode laser [MMOptics, São Carlos, SP, Brazil] was used.

The root canals were obturated with AH plus sealer [Dentsply,De Trey,Konstanz,Germany] and gutta-percha [Dentsply-Maillefer,Ballaigues,Switzerland] using a cold lateral condensation technique. Following root canal obturation, the teeth were permanently restored with composite resin in all the groups.

## Post operative maintenance

Analgesics and antibiotics were prescribed. Both clinically and radiographically post endodontic evaluation was done at 3 month, 6 months, 12months, and 18 months.

- Statistical analysis 

The statistical analysis was performed using a commercially available software [SPSSs 11, SPSS Inc., Chicago, IL, USA]. The study was designed for testing superiority of the disinfection modalities in the root canal. The primary outcome variable was measured using PAI index and clinical parameters. The X2test was used to com-pare the data from baseline to those at3, 6, 12 and 18months for each treatment group and to analyze the statistical significance between the three groups. The significant level was set at p<0.05. Kruskal-Wallis Test was done to to compare the efficacy of each intervension.

## Results

**Figure 2:**
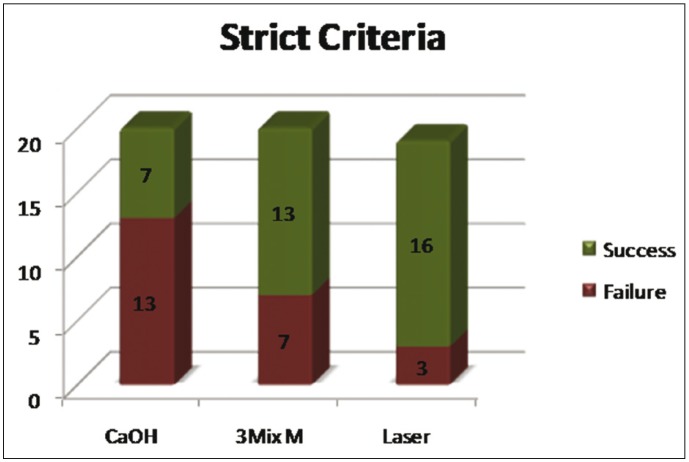
Strict criteria of success between number of patients and root canal studied.

**Figure 3:**
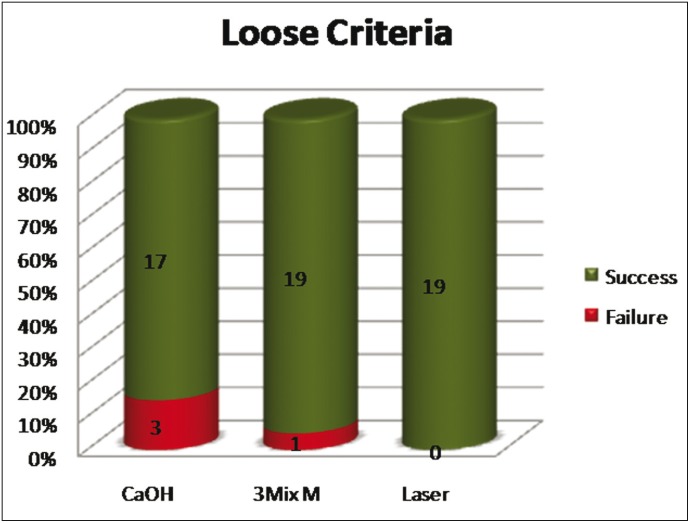
Loose criteria of success between number of patients and root canal studied.

A total of 60 patients fulfilled the inclusion criteria and were included in the study. The patients were selected with a preoperative score of 4 or 5. There were no significant differences for the PAI Scores between the three groups at the start of the experiment ( Table 2). At three month follow up there was no significant change in the PAI score. The radiographic changes for group 1 (Fig. 1) group 2 (Fig. 1) group 3 (Fig. 1). Significant changes in the p value [0.029] occurred at 6 months with 80% of PAD group having a PAI score of 3. However only 30% and 45% of Calcium hydroxide group and triple antibiotic group respectively moved to PAI score of 3. At 12 month one patient in PAD group had dropped out from the study. The remaining 19 patients had lowered their PAI score to 3 from their initial PAI scores of 4 and 5. The PAI score in triple antibiotic paste changed to 3 in 95 % of patients, after 12 months. At 18 months follow up 15 % of cases failed in calcium hydroxide group, 5% in triple antibiotic paste and no failure cases were seen in PAD group. Success criteria were divided into strict and loose, while the former had statistically significant p value the latter did not. (Fig. 2,3). Kruskal-Wallis Test showed an increased mean value for PDT and a significant change in p value. ( Table 3) Bonferroni post hoc test was done to compare if there is any significant change between groups. Only significant change was found between calcium hydroxide and photoactivated disinfection.

**Table 2:**
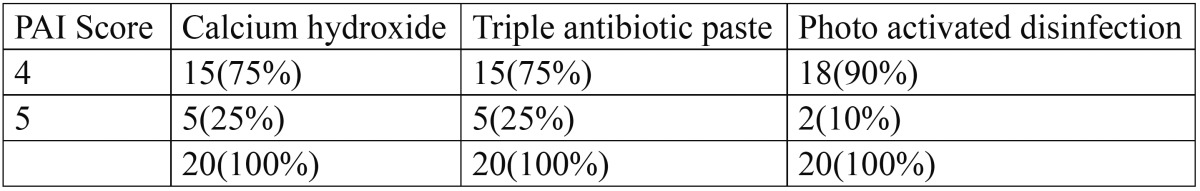
Preoperative.

**Table 3:**
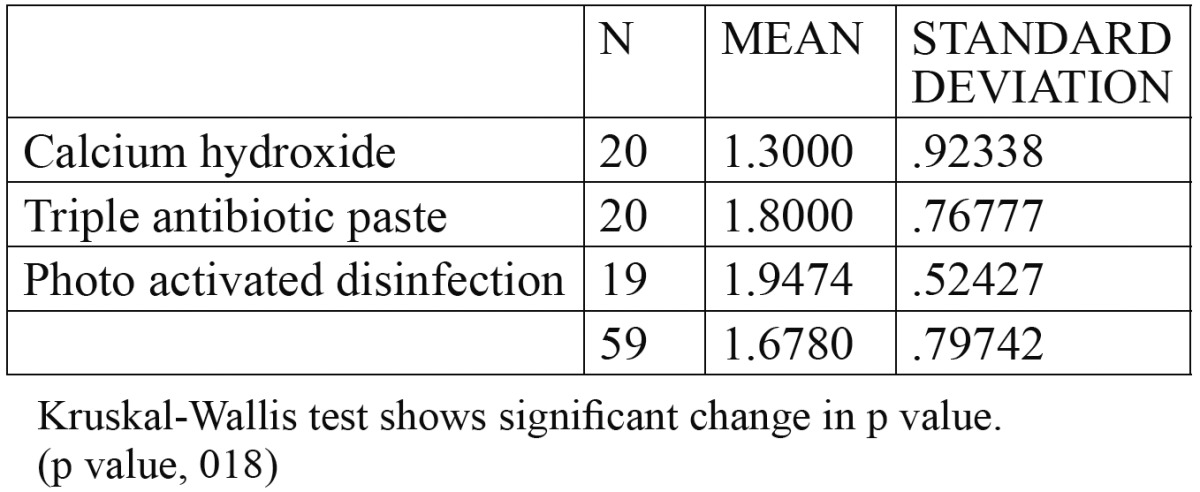
Kruskal-Wallis Test.

## Discussion

Calcium hydroxide is widely used as an intracanal medicament to eliminate microbes in infected root canals (14). Since its introduction by Hermann in 1920, calcium hydroxide has been widely used in endodontics. It is a strong alkaline substance, which has a pH of approximately 12.5. In an aqueous solution, calcium hydroxide dissociates into calcium and hydroxyl ions. Souza et al., suggested that the action of calcium hydroxide beyond the apex may be four-fold: [a] anti-inflammatory activity, [b] neutralization of acid products, [c] activation of the alkaline phosphatase, and [d] antibacterial action (15). By these mechanisms the bacteria in inaccessible areas of the root canal system are killed enabling in the periapical healing. However, there was no increase in antimicrobial effect of calcium hydroxide when left for longer periods in the root canal because the hydroxyl ions do not pass through patent dentinal tubules to alkalize the medium surrounding the teeth (16). The high alkaline pH cannot be maintained within the dentinal tubules because of the buffering effect of dentin (17). A success rate of 80.8 (18) and 73.8% (19) has been reported with calcium hydroxide, when used for endodontic treatment of teeth with periapical lesions. In our study after an eighteen month follow up 85 % success was noted. Clinical evaluation did not prove to show any difference among the three groups,in contrast radiographically there was a change. Loose criteria of success [which includes incomplete success and complete success as successful outcome] showed no difference in success among the groups. Evaluating the success by means of strict criteria the percentile of success is reduced to 35% in calcium hydroxide group. However it is the most common, economical and readily available intracanal medicament.

Research with topical antibiotics has shown that a combination of metronidazole, ciprofloxacin, and minocycline is effective in vitro at killing common endodontic pathogens from necrotic/infected root canals (9). This antibiotic combination is also an effective disinfectant in vivo (20). However, caution should be taken when giving local or systemic drugs. Although the volumes of the drugs applied in this therapy were small, and there were no reports of side-effects, care should be taken if patients are sensitive to chemicals or antibiotics. Minocycline binds to calcium ions via chelation to form an insoluble complex which can cause discoloration of tooth (21). The disadvantage of tooth discoloration induced by minocycline. can be overcome by Cefaclor and fosfomycin (10).This paste cannot be used in patients who respond positively to an allergic patch test to any of the components. The preparation of the drug combination is not time consuming .It is able to address a diverse amount of root canal flora with little chance of resistance .There was a marked reduction in the PAI score from 3 months to 18 months, with 60% of cases having a PAI score of 2.Clinical criteria also suggests an equal success percentile when comparing triple antibiotic paste and photo activated disinfection. According to strict criteria 13 were successful among the 20 cases in this study; this was much higher than the calcium hydroxide group.

Successful killing of S. mutans and E. faecalis by PAD using either methylene blue or tolonium chloride dyes has been reported, with kills of between 97–99.9% for planktonic bacterial loads of up to 10 million organisms. using an exposure time of I20 seconds (22). Also when used in the root canal environment,more consistent ki-lling is seen with tolonium chloride than with methylene blue (22). A key property of photosensitisers to be used with the root canal environment is that they should absorb laser light in the middle red portion of the visible spectrum, since these wavelengths of light give the greatest penetration of dentine and can also penetrate any blood that may be present (23). Middle red wavelengths will also exert direct effects on Gram-negative anaero-bes. The work of Lee (22) demonstrated that the killing effect obtained is greater when the laser light is delivered using a flexible endodontic diffuser which reaches to within 4 mm of the apex and gives even irradiation of the root canal system, rather than a bare optical glass fibre. These results obtained in our study undoubtedly indicate the use of an optical fiber to improve the irradiation in root canals. The fiber probably distributes homogeneously the light inside the root canal guaranteeing a better photoreaction; also, the technique of irradiation using helicoid movements may have contributed to the results.

PAD is a treatment that can be delivered as an addition to conventional endodontic therapy and produces a remarkable additional reduction in bacterial burden. Moreover it appears that a second PAD treatment is even more effective than the first PAD. The reason for this observation is probably that the recolonization of micro-organisms occurs in a less complex biofilm compared with the initial infection that is probably in a fully develo-ped biofilm. Furthermore, as in the second treatment, the number of viable microorganisms was smaller than in the first treatment; the reactive oxygen species formed during PAD had a bigger chance of producing an irrepa-rable oxidative stress because of the ratio between reactive oxygen species and microorganisms (24). We have employed tolonium chloride as the photosensitizer and a diode laser coupled with an optical fiber as a light source. The use of a chelating agent after instrumentation, in our case EDTA instead of citric acid used by Bon-sor (25), acts as a cleaner and disrupter of the biofilm expanding the access of the tolonium chloride to the canal system.

Our results concluded a 100% success using loose criteria and by clinical valuation.Interestingly there were no failure cases. After 18 months 80% of cases in the PAD group had a PAI score of 2. Whatever means was used for success [clinical and radiographic) PAD proved to be the best among the groups. However, there are several limitations that may be associated with the intra-canal use of laser that cannot be overlooked (26).The emission of laser energy from the tip of the optical fiber or the laser guide is directed vertically along the root canal wall and not necessarily laterally to the root canal walls (27). Thus, it is impossible to obtain uniform coverage of the canal surface using the laser (26,27). It has been recommended in root canal treatment as an alternative or a supplement to currently used disinfection methods (28) .The number of failure cases were less in PAD group because less chances of development of resistant species (29) as compared to other groups Triple antibiotic paste is a cheaper alternative to photo dynamic therapy.

Our study shows there is no difference in the clinical outcome in all the three groups however radiographically there is a statistically significant difference in the strict criteria. This indicates the need for radiographic method for identifying success rather than clinical means. The study concluded that the PAD group was most effective root canal disinfectant and aided in the healing of periapical lesions.
